# Identification and validation of mitophagy and astrocyte-related molecular signature in the pathogenesis of Alzheimer's disease: evidence from ensemble learning-driven multi-omics and clinical validation

**DOI:** 10.3389/fnins.2026.1910621

**Published:** 2026-07-24

**Authors:** Jiacheng Liu, Wei Chen

**Affiliations:** 1 The Seventh Clinical Medical College, Anhui University of Chinese Medicine, Fuyang, Anhui, China; 2 Department of Neurology II, The Seventh Affiliated Hospital of Anhui University of Chinese Medicine, Fuyang, Anhui, China

**Keywords:** Alzheimer's disease, astrocyte, clinical validation, ITSN1, mitophagy, multi-omics

## Abstract

**Background:**

Alzheimer's disease (AD) is a progressive neurodegenerative disorder with limited diagnostic tools and therapeutic options. Dysregulated mitophagy in astrocytes plays a pivotal role in AD pathogenesis. This study aims to identify a mitophagy and astrocyte (MA)-associated molecular signature for AD diagnosis and therapeutic targeting.

**Methods:**

Limma, WGCNA, xCell, PPI network and integrated machine learning pipeline coupled with SHAP were deployed on AD patient hippocampal bulk profiles (GSE28146, GSE36980, GSE29378, GSE48350) for identification of MA-associated predictive model and hub gene. Next, astrocyte patten and MA-associated hub gene molecular performance were estimated in hippocampal single-cell profile of AD patients (GSE163577) via advanced analytical frameworks. In addition, active learning framework and molecular docking was deployed in GSE29378 for identification of therapeutic candidate for AD patients by targeting MA-associated hub gene. Furthermore, AD hippocampal tissues were collected, and then MA-associated hub gene expression was estimated.

**Results:**

A core 8-gene MA signature (ITSN1, VLDLR, CYP7A1, SREBF2, RASL12, TPMT, CYP4X1, ARHGEF) was identified, which can guide the molecular subgroup identification and predictive model construction for AD patients. ITSN1 can be considered as the MA-associated hub gene in AD pathogenesis, which was up-regulated and predominantly expressed in astrocytes. Drug repositioning identified BRD-K10008415 as the potential compound predicted to reverse the AD signature by targeting ITSN1.

**Conclusions:**

This study identified ITSN1 as a MA-associated critical hub potential connecting mitophagy dysregulation and astrocyte dysfunction in AD. We also identified MA-associated molecular signatures that can potentially elaborate predictive effects on AD pathogenesis. BRD-K10008415 can be considered as potential candidate for AD treatment by targeting ITSN1.

## 1 Introduction

Alzheimer's disease (AD) is the most prevalent neurodegenerative disorder, affecting an estimated 50 million individuals worldwide, a figure projected to triple by 2050 (Lane et al., 2018). Its hallmark neuropathological features include extracellular amyloid-beta (Aβ) plaques and intracellular neurofibrillary tangles of hyperphosphorylated tau, leading to progressive synaptic failure and neuronal loss (Lopez-Lee et al., 2024). The hippocampus, a brain region critical for memory consolidation, is among the earliest and most severely affected structures in AD, exhibiting pronounced atrophy and metabolic dysfunction (Hardy, 2025). Epidemiological studies have linked risk factors such as aging, the APOE ε4 allele, and vascular comorbidities to AD, yet the precise molecular cascades driving its relentless progression remain incompletely understood (Rostagno, 2022). Consequently, there is an unmet need for novel diagnostic biomarkers and disease-modifying therapies.

Mitophagy, a selective form of autophagy that orchestrates the removal of damaged mitochondria, is essential for maintaining neuronal homeostasis (Picca et al., 2023). In AD, mitochondrial dysfunction is an early and invariant feature, with accumulated defective mitochondria contributing to oxidative stress, bioenergetic failure, and calcium dysregulation (Fang et al., 2019). Indeed, post-mortem AD brains exhibit decreased expression of mitophagy receptors like BNIP3L/NIX and FUNDC1, alongside accumulation of autophagic vacuoles (Kerr et al., 2017). Parallel to these neuronal deficits, mounting evidence implicates a central role for reactive astrocytes in AD pathology (Ziar et al., 2025). Astrocytes, once viewed merely as support cells, are now recognized as active participants in neuroinflammation, synaptic pruning, and metabolic support (Andersen et al., 2022). In AD, astrocytes undergo reactive gliosis, losing their neuro-supportive functions and gaining neurotoxic properties, often through altered lipid metabolism and glutamate handling (Serrano-Pozo et al., 2024). The interplay between astrocyte dysfunction and mitophagy failure, however, remains a critical gap in our understanding of AD pathogenesis.

To bridge this divide, we elucidated that a shared molecular program linking mitophagy dysregulation and astrocyte underpins AD progression. Here, we conducted an integrated multi-omics analysis of hippocampal bulk and single-cell transcriptomes, coupled with an ensemble machine learning framework, to identify a core MA-associated molecular signature in AD. Our objectives were to build a robust diagnostic model, pinpoint a central hub gene, characterize its cellular specificity and functional role, and identify a candidate therapeutic compound for AD patients.

## 2 Material and methods

### 2.1 Source of bulk profiles

Bulk RNA-sequencing datasets derived from human hippocampal tissue were retrieved from the GEO database. GSE36980 (internal set 2) included 8 AD and 10 control samples (Liu et al., 2024). GSE28146 (internal set 1) comprised 8 control and 22 AD samples (Wang et al., 2022). GSE29378 (training set) contained 32 control and 31 AD samples, while GSE48350 (validation set) included 43 control and 19 AD samples (Prabha et al., 2024; Liu et al., 2024). All datasets were normalized by sva package in R (Leek et al., 2012). Mitophagy-related gene list was acquired from GeneCards database with relevance threshold more than 1.

### 2.2 Identification of DEGs via Limma analysis

Differential expression analysis between AD and control samples in GSE28146 (internal set 1) was conducted using the Limma package in R (Liu et al., 2021). Genes satisfying an adjusted *p*-value < 0.05 and |log_2_ (Fold Change)| > 0.5 were defined as DEGs (Ritchie et al., 2015). The intersection of DEGs with mitophagy-related gene list yielded mitophagy-associated DEGs. Their expression pattern was visualized in a heatmap using the ComplexHeatmap package in R (Gu, 2022). Functional enrichment (GO and KEGG) was performed using the clusterProfiler package in R, referencing the KEGG and GO gene set from MSigDB database (Yu et al., 2012).

### 2.3 xCell and WGCNA analysis for co-expression module identification

The xCell R package was used to infer the relative abundance of 64 immune and stromal cell types, including astrocytes, in GSE36980 (internal set 2; Aran et al., 2017). To identify co-expression networks associated with AD and astrocyte scores, we performed a WGCNA using the WGCNA package in R (Langfelder and Horvath, 2008). A scale-free topology model was built using a soft-thresholding power (β) selected based on an *R*^2^ > 0.85 (Langfelder and Horvath, 2008). The adjacency matrix was converted into a topological overlap matrix (TOM), and modules were identified via hierarchical clustering with a minimum module size of 60 (Langfelder and Horvath, 2008). The Pearson correlation between module eigengenes and both the AD phenotype and the astrocyte enrichment score was calculated (Langfelder and Horvath, 2008). The module showing the highest positive correlation with both traits was designated as the key module (Langfelder and Horvath, 2008). Genes within this module were then intersected with the previously identified mitophagy-associated DEGs to define the MA-associated shared DEGs.

### 2.4 Integrated machine learning pipelines for diagnostic model construction

For MA-associated shared DEGs, we performed STRING database and MOCODE plugin screening in Cytoscape software for identification of MA-associated crucial DEGs (Szklarczyk et al., 2023; Shannon et al., 2003). Next, based on MA-associated DEGs, 132 distinct machine learning combinations were generated by pairing 11 classification algorithms with 12 feature selection methods in GSE29378 and GSE48350 (Wu et al., 2024). Briefly, the 132-model machine learning framework typically represents a comprehensive grid search approach that systematically combines multiple classification algorithms with various feature selection methods and preprocessing strategies. Combinations of these algorithms were applied to the MetaGSE for signature selection and model construction using 10-fold cross-validation repeated 3 times (Wu et al., 2024). The optimal model was selected based on the highest mean area by achieving high AUC-index (Wu et al., 2024). The final model performance was externally validated using GSE48350 (Wu et al., 2024). ROC and PR curves were generated in GSE29378 and GSE48350 via pROC package in R (Robin et al., 2011). A nomogram and calibration curve were plotted using the rms package in R to assess the model predictive accuracy and clinical utility (Lin et al., 2024). To interpret model output, the SHAP algorithm in R was applied to quantify each gene contribution to the final diagnosis, with the top contributor designated the central hub gene (Zuo et al., 2023).

### 2.5 Consensus clustering for molecular subgroup identification

Based on the expression levels of the identified MA-associated crucial shared DEGs, an unsupervised consensus clustering was performed on the GSE29378 training set AD samples using the ConsensusClusterPlus package in R (Zhou et al., 2026). The optimal number of clusters (k) was determined by evaluating the consensus cumulative distribution function (CDF) curves, delta area plots, and tracking plots (Zhou et al., 2026). Expression differences in MA-associated crucial shared DEGs between different subgroups were estimated and visualized by complexheatmap package in R (Zhou et al., 2026). Pathway enrichment between subgroups, were estimated by GSEA analysis using the clusterProfiler package in R, referencing the KEGG and GO gene set from MSigDB database (Zhou et al., 2026). Immune cell infiltration between subgroups was estimated via CIBERSORT package in R (Zhou et al., 2026).

### 2.6 Single-cell analysis

The single-cell RNA-seq dataset GSE163577, derived from hippocampal tissue of 9 AD patients, was processed using the Seurat package in R (Tan et al., 2023). Quality control (QC) involved filtering cells with feature counts between 200 and 5,000 and a mitochondrial gene percentage less than 10% (Tan et al., 2023). Data were normalized using the SCTransform method. PCA was performed for dimensionality reduction, followed by graph-based clustering and visualization via UMAP and t-SNE (Tan et al., 2023). Cell types were annotated using the SingleR package in R with the Human Primary Cell Atlas reference (Cao et al., 2023). Cell-cell communication among identified clusters was analyzed using CellChat package in R (Fang et al., 2022). Metabolic heterogeneity was assessed using the scMetabolism package in R (Qing et al., 2024). The activity of the mitophagy pathway was calculated using the AUCell package in R, scoring each cell based on the curated mitophagy gene set (Deng et al., 2021). Expression of the hub gene across cell types was visualized on a dot feature plot. An *in silico* knockout of the hub gene specifically in astrocytes was simulated using the scTenifoldKnk package in R (Osorio et al., 2022). The top 10 perturbed genes were identified and subjected to GENEMINIA analysis and GO and KEGG enrichment using clusterProfiler package in R, referencing the KEGG and GO gene set from MSigDB database (Ma, 2026). Differentiation trajectories of astrocytes were inferred using the Monocle2 package in R, with hub gene expression mapped along the pseudotime trajectory (Huang et al., 2024).

### 2.7 Drug prediction analysis

To identify potential therapeutic agents active against the hub gene, we employed the active learning platform in R (Chen et al., 2026). Using the differentially expressed genes between AD and control in GSE29378 as input, the platform integrated this signature with the cMAP drug perturbation database (Chen et al., 2026). Each compound was assigned a Drug-Target Association Score (DTAS) based on the connectivity of its gene expression signature to the query signature (Chen et al., 2026). The compound with the highest DTAS was selected (Chen et al., 2026). Its binding affinity with the hub gene product was then validated via molecular docking using AutoDock Vina software (Chen et al., 2026). The crystal structure of the hub protein was retrieved from the PDB Database, and the compound structure was downloaded from PubChem database (Chen et al., 2026). Binding energies were calculated, and the lowest energy conformation was visualized using PyMOL software (Chen et al., 2026).

### 2.8 Clinical validation

To validate the mRNA expression of the hub gene identified in the bioinformatics analysis, we collected hippocampal tissue samples from 6 AD patients and 6 age-matched cognitively normal controls. All samples were obtained with ethical approval and written informed consent from the donors or their legal representatives. Fresh hippocampal tissue (~50 mg per sample) was immediately dissected, snap-frozen in liquid nitrogen within 30 min of collection and stored at −80 °C until RNA extraction. Each frozen sample was homogenized in 1 mL of TRIzol reagent (Invitrogen, Cat# 15596026) using a Precellys 24 tissue homogenizer (Bertin Technologies) at 6500 rpm for 2 × 20 s with a 10 s interval, keeping samples on ice throughout to minimize RNA degradation. Total RNA was isolated according to the manufacturer's protocol for TRIzol. 1 μg of total RNA from each sample was reverse-transcribed into cDNA using the PrimeScript RT Reagent Kit with gDNA Eraser (Takara) following the manufacturer's instructions. The reaction was performed in a T100 Thermal Cycler (Bio-Rad) under the following conditions: 42 °C for 2 min (gDNA removal), 37 °C for 15 min, then 85 °C for 5 s (enzyme inactivation). The relative expression level of ITSN1 was calculated using the 2^−^Δ*ΔCt* method, with GAPDH as the endogenous control. q-RT-PCR was carried out using TB Green Premix Ex Taq II (Tli RNaseH Plus) (Takara).The primer sequences for ITSN1 were:

ITSN1:
F:5′-CCGAAGTCATCA CCTACAGCC-3′,
R: 5′-GGAGCAGATGGCGATACAGA-3′;
GAPDH:
F:5′-GG GCGAGATCCCTCCAAAAT-3′,
R: 5′-GGCTGTTGTCATACTTCTCATGG-3′.

### 2.9 Statistical analysis

All statistical analyses were performed using R version 4.2.2 or GraphPad Prism version 9.1. Comparisons between two groups were conducted using an unpaired Student's *t*-test for normally distributed data or a Mann-Whitney U test for non-normally distributed data. Correlation coefficients were calculated using Spearman's rank method. A *p*-value < 0.05 was considered statistically significant.

## 3 Results

### 3.1 Identification of mitophagy-related DEGs for AD patients

**Figure 1:**
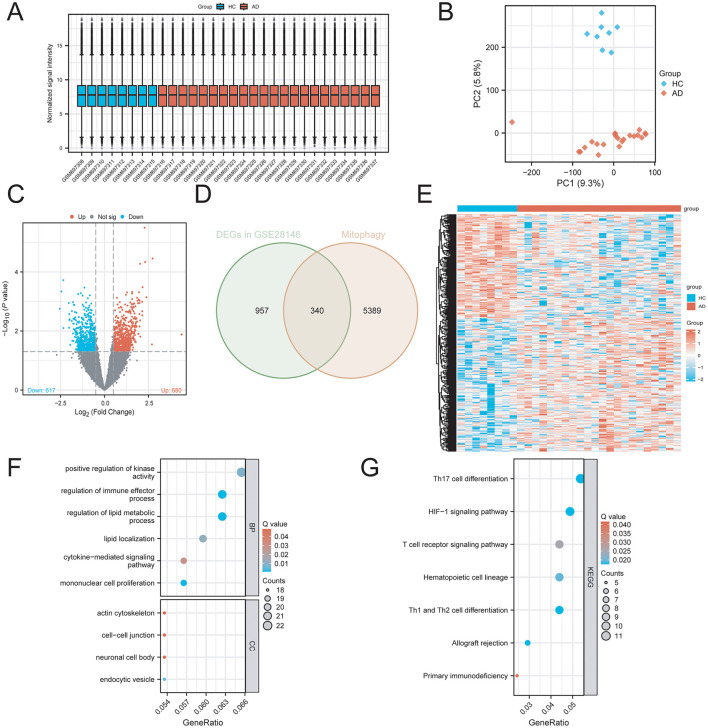
Identification of mitophagy-associated DEGs in GSE28146. **(A)** Box plot of GSE28146 after normalization. **(B)** PCA plot showing separation between AD and control samples. **(C)** Volcano plot of DEGs. **(D)** Venn diagram depicting the intersection of DEGs and mitophagy genes, yielding 340 mitophagy-associated DEGs. **(E)** Heatmap of the 340 mitophagy-associated DEGs. **(F, G)** GO and KEGG enrichment analysis.

In the GSE28146 dataset, differential expression analysis between hippocampal AD and control samples yielded 1,297 DEGs (Figures 1A–C). The intersection of these genes with the curated mitophagy gene list resulted in 340 mitophagy-associated DEGs (Figure 1D). A heatmap of these 340 genes revealed a distinct expression pattern that clearly separated AD samples from controls (Figure 1E). Enrichment analysis of these mitophagy-associated DEGs revealed a strong association with biological processes related to lipid metabolism, such as lipid metabolism and immune-related pathways (Figures 1F, G).

### 3.2 Identification of MA-associated shared DEGs for AD patients

**Figure 2:**
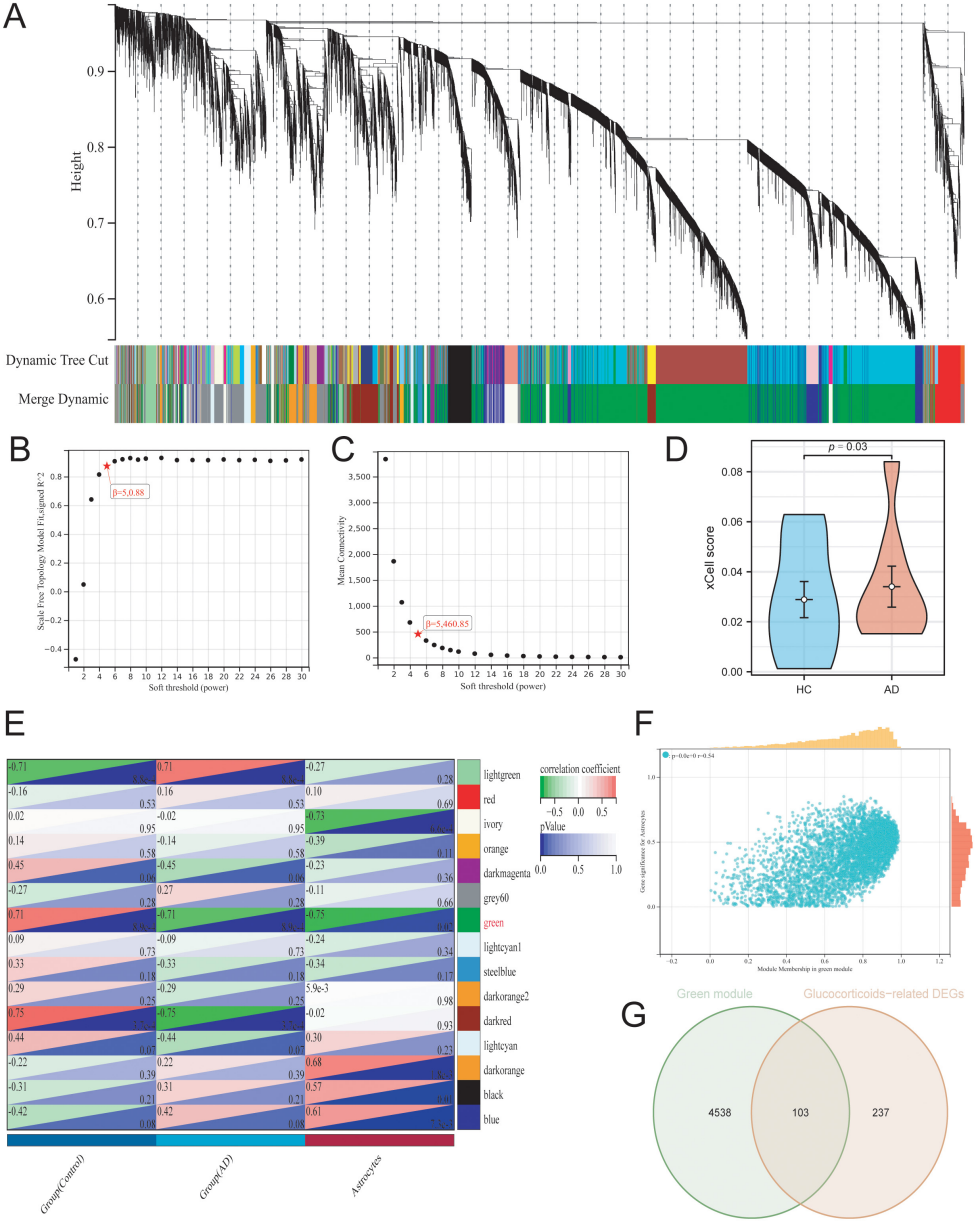
Identification of MA-associated shared DEGs in GSE36980. **(A–C)** Scale-free topology fit index and mean connectivity analysis for soft-threshold power selection. **(D)** Box plot showing astrocyte enrichment scores in AD compared to control from xCell analysis. **(E, F)** Module-trait relationship heatmap, highlighting the green module with the highest correlation to AD and astrocyte scores. **(G)** Venn diagram of the intersection between green module genes and mitophagy-associated DEGs, yielding 103 shared MA-associated DEGs.

During WGCNA, hierarchical clustering was first performed to detect outlier samples (Figure 2A), followed by selection of the soft-thresholding power based on the scale-free topology fit index and mean connectivity analyses (Figures 2B, C). Analysis of cellular composition in GSE36980 using xCell algorithm indicated a significantly higher enrichment score for astrocytes in AD patients compared to controls (Figure 2D). WGCNA was then performed on this dataset. A scale-free topology fit index (*R*^2^) of soft-threshold power =5,0.88 and 5,460.85 was chosen to achieve a scale-free topology fit index (Figures 2B, C). The analysis identified several co-expression modules. The green module showed the strongest positive correlation with both the AD phenotype and the astrocyte enrichment score (Figures 2E, F). Extracting the genes from this green module and intersecting them with the 340 mitophagy-associated DEGs yielded a set of 103 MA-associated shared DEGs (Figure 2G).

### 3.3 MA-associated predictive model construction for AD patients

**Figure 3:**
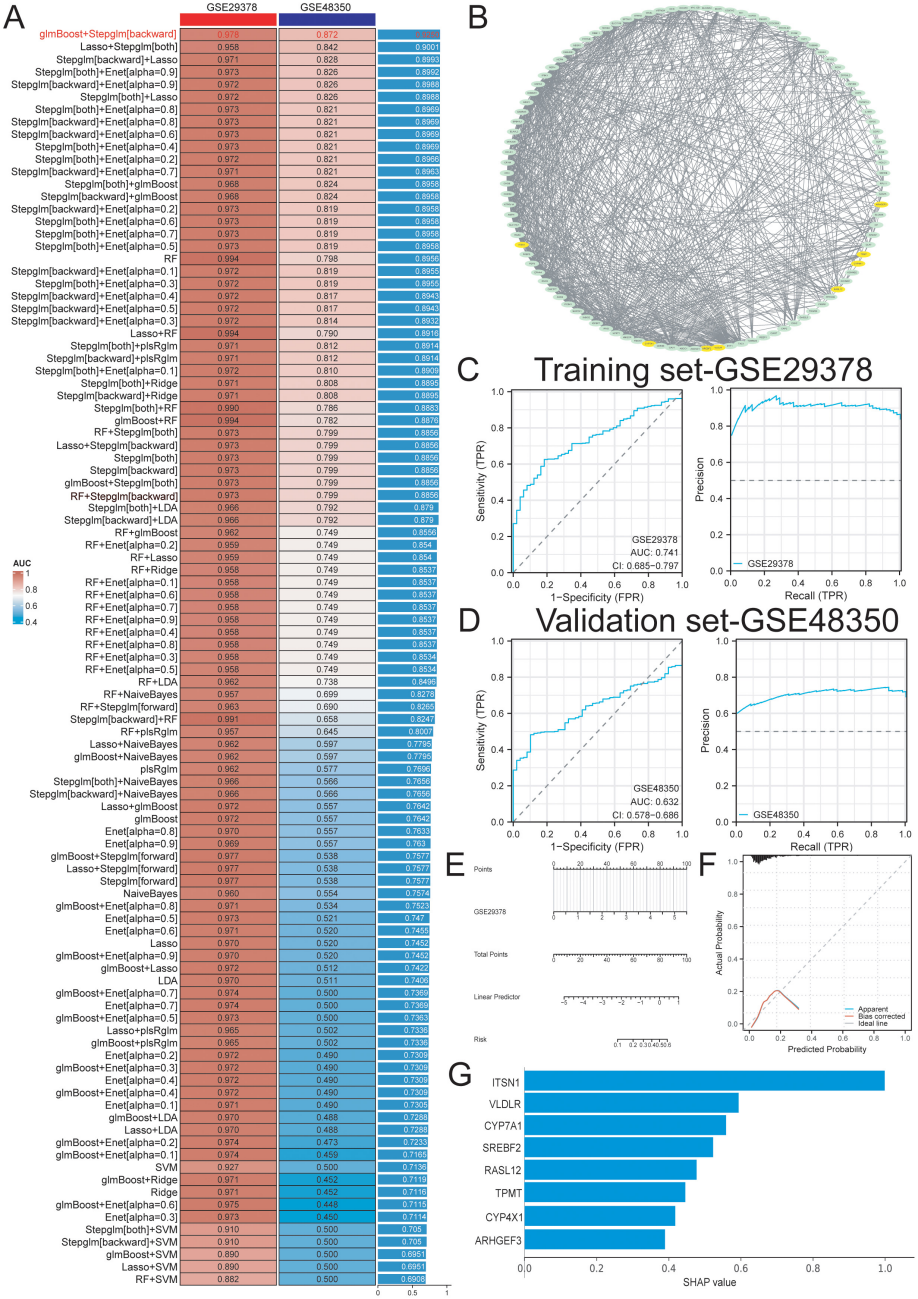
MA-associated diagnostic model construction for AD patients. **(A)** Performance ranking of 132 machine learning models, with the glmboost+stepglm model highlighted. **(B)** PPI network of 8 core genes (STRING/MOCODE). **(C, D)** ROC curves in the GSE29378 training set and GSE48350 validation set. **(E, F)** Nomogram and calibration in training and validation sets. **(G)** SHAP summary plot showing ITSN1 as the top contributor.

Protein-protein interaction analysis of the 103 MA-associated DEGs was performed using STRING, and hub genes were identified using the MOCODE algorithm in Cytoscape, revealing 8 core genes, including ITSN1, VLDLR, CYP7A1, SREBF2, RASL12, TPMT, CYP4X1, and ARHGEF (Figure 3B). An ensemble of 132 machine learning models was then trained on the GSE29378 dataset (Figure 3A). The model combining glmboost with stepglm (backward) achieved the highest mean AUC-index of 0.9250 across cross-validation folds, and was selected as the optimal model (Figure 3A). This model demonstrated robust diagnostic performance with an AUC of 0.741 in the GSE29378 training set (Figure 3C) and an AUC of 0.632 in the GSE48350 validation set (Figure 3D). Nomogram and calibration confirmed model high sensitivity and specificity (Figures 3E, F). SHAP analysis ranked ITSN1 as the most influential feature among MA-associated shared DEGs, contributing the most to the diagnosis prediction (Figure 3G).

### 3.4 MA-associated molecular subgroup identification for AD patients

**Figure 4:**
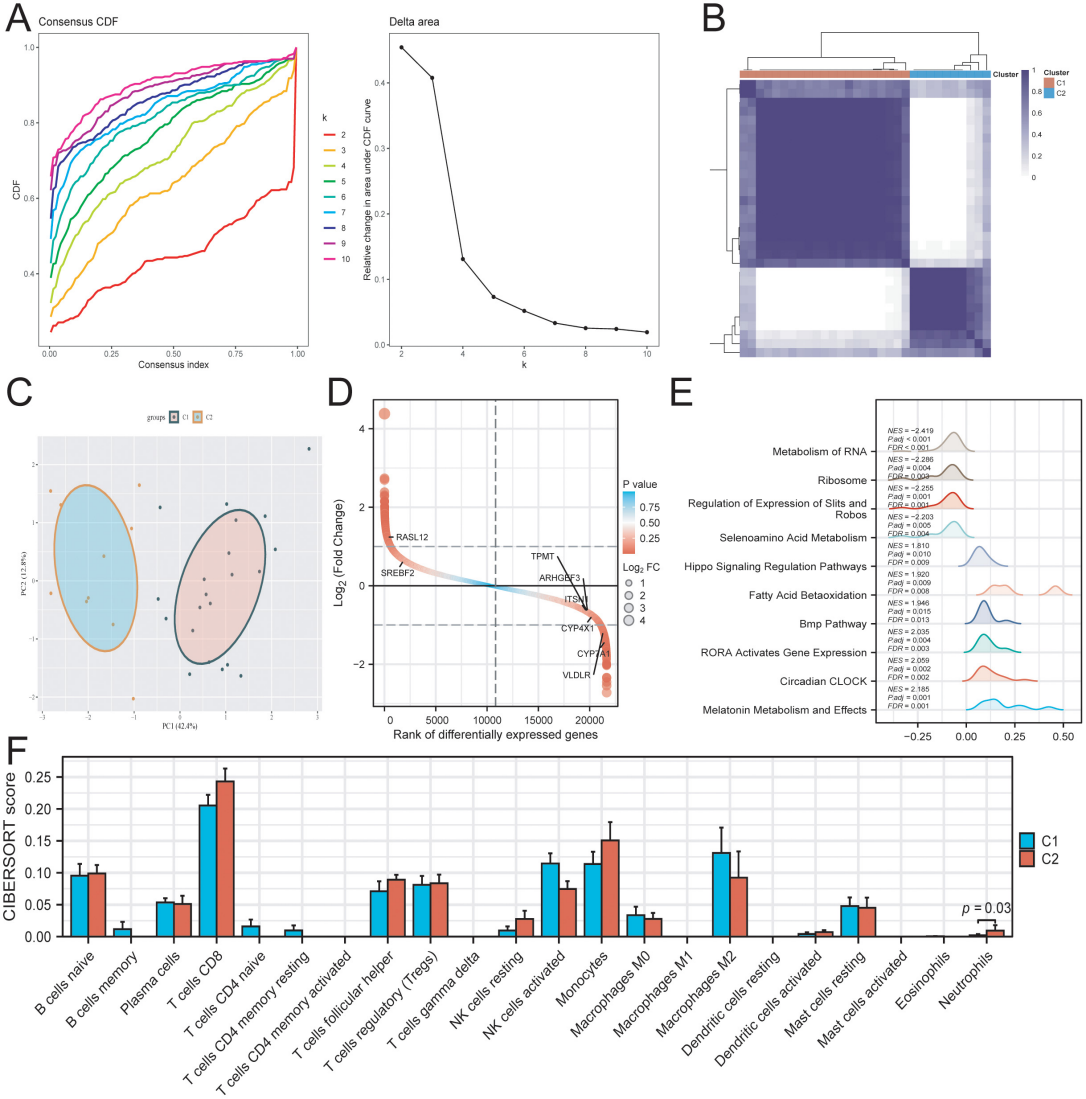
Molecular subgroups based on MA signature for AD patients. **(A, B)** Consensus clustering CDF curve and delta area plot. **(C)** PCA plot of different molecular subgroups. **(D)** Heatmap of 8 MA-associated DEG expression in GSE29378 patients. **(E)** GSEA enrichment plots for C1 compared to C2 subgroups. **(F)** Immune cell infiltration (CIBERSORT) differences between C1 and C2.

Consensus clustering of AD patients in GSE29378 based on the 8 MA-associated DEGs identified two distinct molecular subtypes, designated C1 and C2 (Figure 4A–C). GSEA analysis revealed that the C2 subgroup was significantly enriched in pathways related to metabolism of RNA and functions related to mitophagy (Figure 4E). CIBERSORT analysis showed significant differences in immune cell infiltration between the two subtypes, with the C2 cluster exhibiting a higher proportion of activated neutrophil, suggesting a more pro-inflammatory microenvironment (Figure 4F). Heatmap visualization confirmed the distinct expression patterns of the 8 MA-associated DEGs between C1 and C2 (Figure 4D).

### 3.5 Astrocyte implications at single-cell level for AD patients

**Figure 5:**
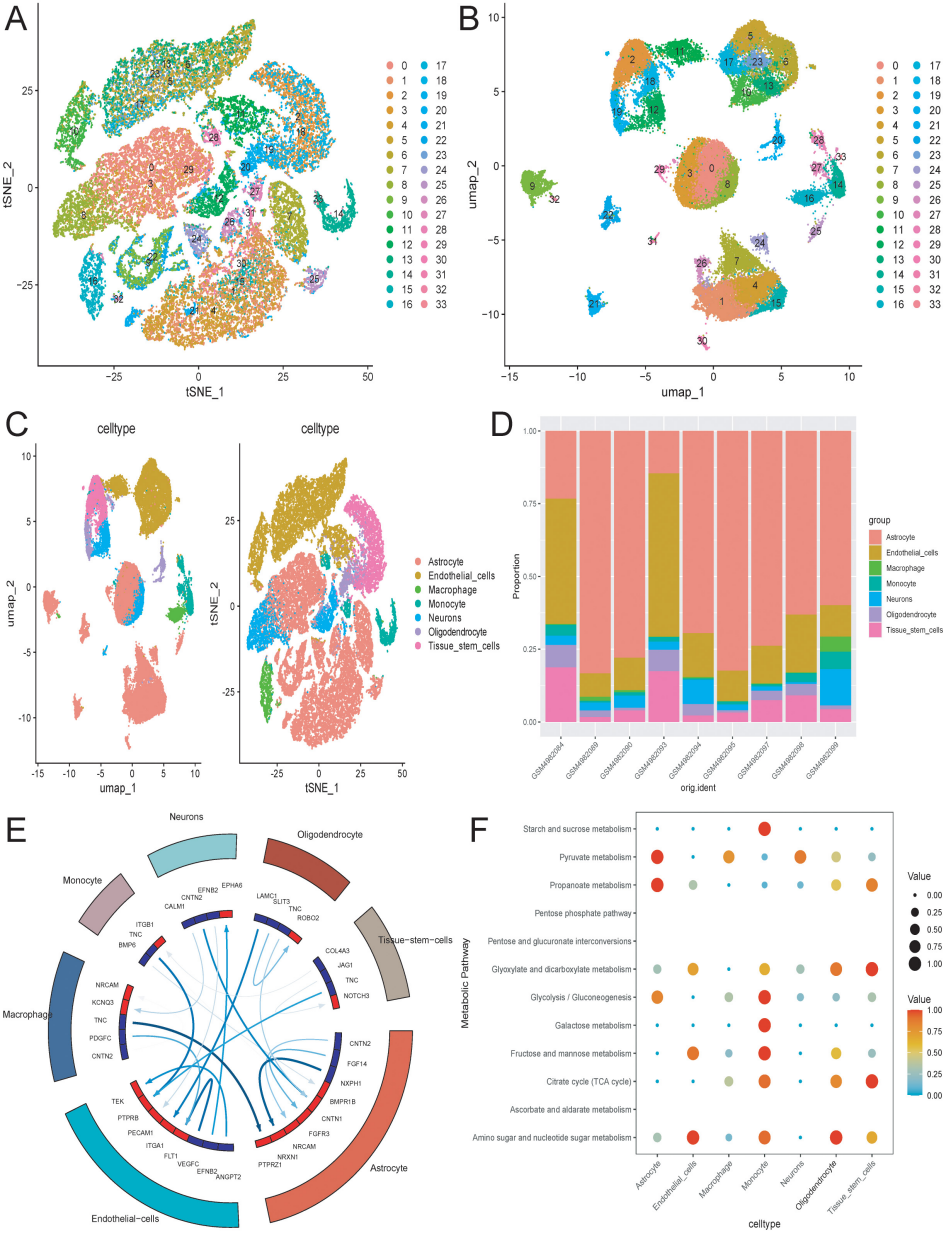
Single-cell landscape of AD hippocampus. **(A)** t-SNE plot showing 33 cell clusters. **(B)** UMAP plot showing 33 cell clusters. **(C)** UMAP and t-SNE plots of 7 annotated cell types. **(D)** Cell proportion estimation across samples. **(E)** CellChat network plot showing astrocyte-centered intercellular communication. **(F)** Dot plot of metabolic pathway activity across cell types.

Single-cell analysis of the GSE163577 hippocampus dataset identified 33 cell clusters post-quality control and dimensionality reduction (Supplementary Figures 1A–D and Figures 5A, B). Using SingleR, these clusters were annotated into 7 major cell types (Figure 5C). Astrocytes constituted the most abundant population (Figure 5D). CellChat analysis revealed that astrocytes engaged in extensive and complex intercellular communication, predominantly with endothelial cells (Figure 5E). Metabolic heterogeneity analysis indicated that astrocytes exhibited distinct metabolic pathway activities, with a notable upregulation of pyruvate metabolism and propanoate metabolism compared to other cell types (Figure 5F).

### 3.6 MA-associated hub gene molecular pattern estimations at single-cell level for AD patients

**Figure 6:**
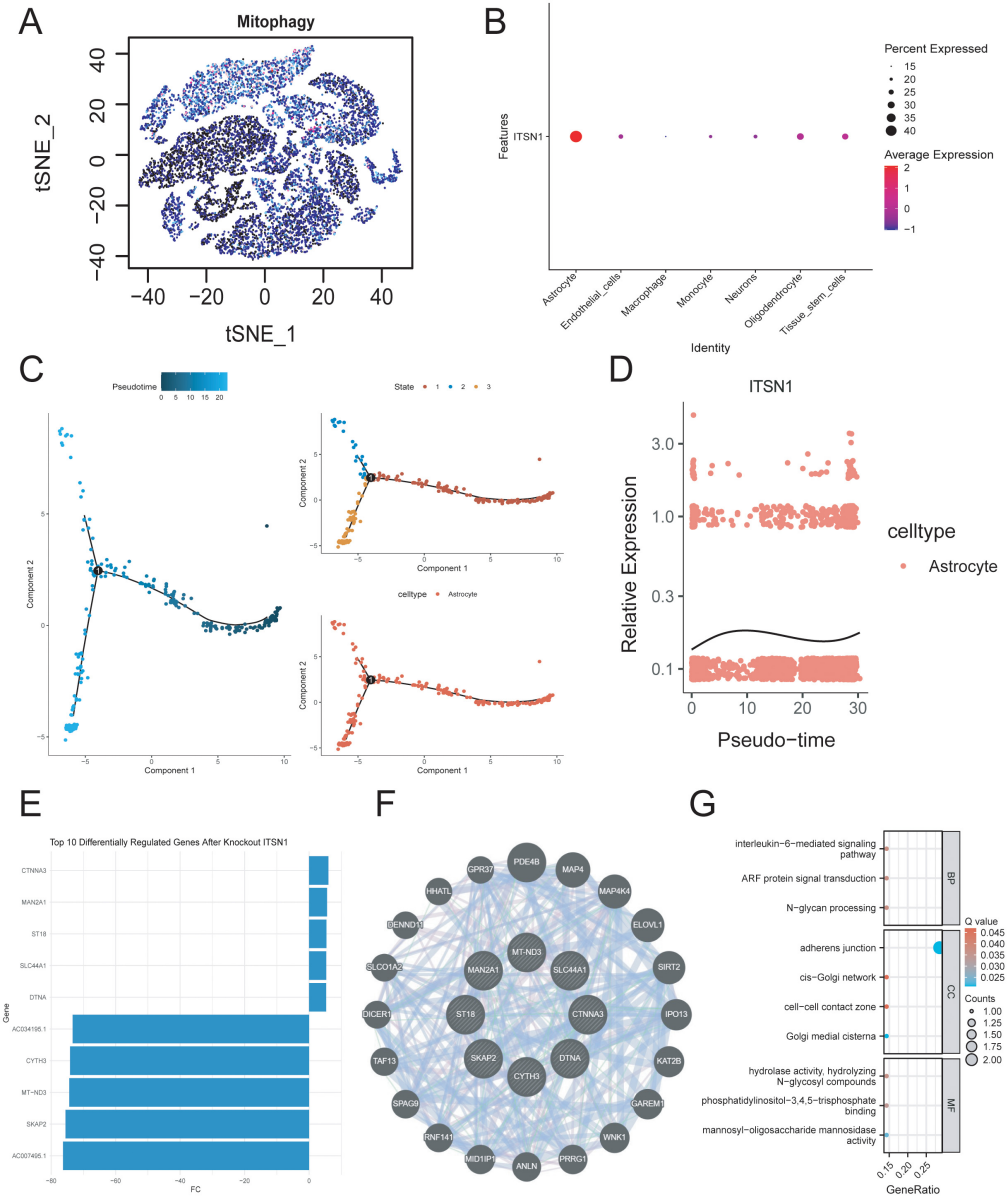
Single-cell characterization of ITSN1 in astrocytes of AD patients. **(A)** AUCell score for mitophagy pathway across cell types, showing highest activity in astrocytes. **(B)** Feature plot showing ITSN1 expression enriched in astrocytes. **(C)** Pseudotime trajectory of all astrocytes, revealing 3 branches. **(D)** ITSN1 expression mapped along the astrocyte pseudotime. **(E–G)** *in silico* ITSN1 knockout and corresponding functional analysis.

AUCell scoring of mitophagy pathway activity showed the highest activation in astrocytes (Figure 6A). Similarly, ITSN1 expression was predominantly localized to the astrocyte cluster, with very low expression in other cell types (Figure 6B). Virtual knockout of ITSN1 specifically in astrocytes via scTenifoldKnk identified perturbed ISTN1 predicted a disruption of pathways related to mitophagy (Figures 6E–G). Pseudotime trajectory analysis of all astrocytes using Monocle2 revealed three distinct differentiation branches (Figure 6C). Mapping ITSN1 expression along this pseudotime revealed that its expression levels were high and stable across the main astrocyte trajectory, suggesting a fundamental role in astrocyte function (Figure 6D).

### 3.7 Validation of MA-associated hub gene expression and corresponding therapeutic enrichment

**Figure 7:**
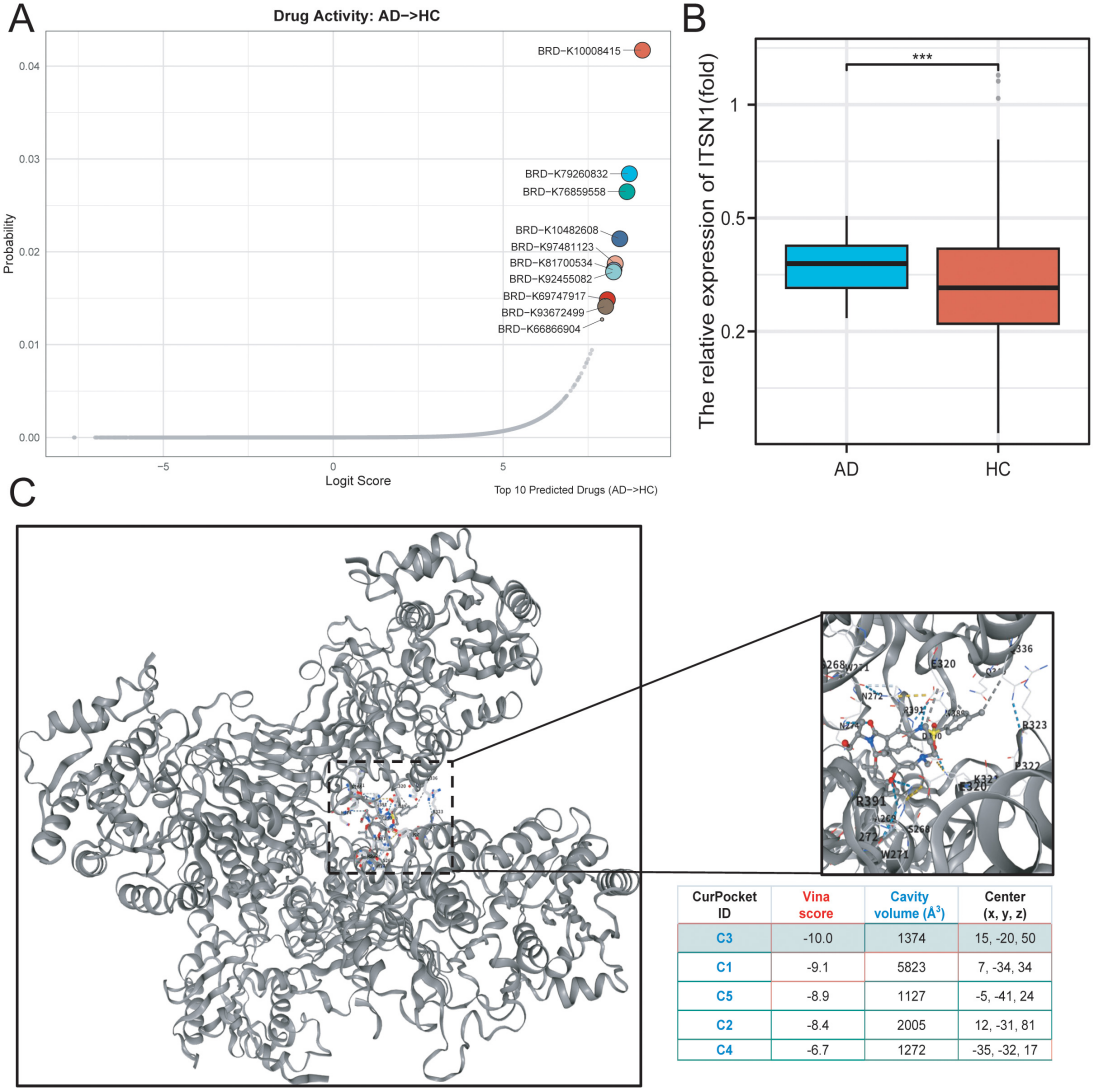
Drug prediction and clinical validation targeting ITSN1 for AD patients. **(A)** DrugReflector ranking of compounds, showing BRD-K10008415 as top-ranked. **(B)** q-RT-PCR validation of ITSN1 protein expression in AD compared to control human hippocampal tissue. **(C)** Molecular docking estimation between BRD-K10008415 and ITSN1. The symbol *** indicates *p* < 0.001.

Drug repositioning using DrugReflector identified BRD-K10008415 as the top-ranked compound predicted to reverse the AD-associated gene expression signature (Figure 7A). Molecular docking analysis between BRD-K10008415 and the protein structure of ITSN1 predicted a strong binding affinity with a binding energy of −10.0 kcal/mol, suggesting a high potential for physical interaction (Figure 7B). Finally, clinical validation in our collected hippocampal samples confirmed that ITSN1 protein levels were significantly elevated in AD patients compared to controls, thereby substantiating our bioinformatics findings (Figure 7B). Molecular docking analysis between BRD-K10008415 and ITSN1 predicted a strong binding affinity with a binding energy of -10.0 kcal/mol (Figure 7C).

## 4 Conclusion and discussion

In this study, we conducted a comprehensive multi-omics investigation integrating bulk transcriptomics, single-cell analysis, and ensemble machine learning to delineate a novel MA-associated molecular signature in AD. We identified an 8-gene MA-associated signature potentially capable of stratifying AD patients into distinct molecular subtypes and constructed a high-performance diagnostic model. Our central finding is the identification of ITSN1 as the pivotal hub gene potential connecting mitophagy dysregulation and astrocyte dysfunction in AD.

ITSN1 (Intersectin 1) is a multi-domain scaffolding protein known to be critical for clathrin-mediated endocytosis, a process tightly linked to mitophagy (Bruel et al., 2022). The PINK1/Parkin pathway, the canonical mitophagy cascade, relies on the displacement of damaged mitochondrial fragments into autophagosomes, a process that is endocytosis-like (Keating et al., 2006). Previous work has shown that ITSN1 interacts with dynamin and other endocytic machinery to regulate mitochondrial dynamics and autophagosome formation (Mintoo et al., 2024). Our findings that *in silico* knockout of ITSN1 in astrocytes perturbs the mitophagy pathway directly aligns with this literature, suggesting that ITSN1 is a key regulator of mitochondrial quality control in these cells (Spargo et al., 2025). In the context of astrocytes, ITSN1 has been studied less extensively, but its role is gaining recognition (Spargo et al., 2025). ITSN1 is highly expressed in astrocytes and is crucial for glutamate receptor trafficking and the clearance of synaptic debris, processes vital for maintaining synaptic homeostasis (Shergill and Azarnia Tehran, 2026). Its dysregulation, as observed in our study with elevated ITSN1 levels in AD, may lead to aberrant astrocytic endocytosis, potentially disrupting the clearance of toxic Aβ species and propagating neuroinflammation (Shergill and Azarnia Tehran, 2026). Furthermore, ITSN1 has been genetically linked to late-onset AD in genome-wide association studies (Malakooti et al., 2019). Our data provides functional evidence for this link, showing that ITSN1 is a driver of a maladaptive astrocyte phenotype in AD, characterized by a perturbed mitophagy program and altered metabolic signaling.

Collectively, our results establish MA-associated molecular patterns and identify ITSN1 as a central hub coupling mitophagy dysregulation with astrocyte dysfunction in AD, provide a clinically applicable diagnostic model, and highlight a novel therapeutic target warranting further preclinical investigation. However, several limitations must be acknowledged. The initial transcriptomic findings were obtained from public databases, and despite the application of batch correction, certain technical and biological variabilities may remain unaccounted for. To enhance clinical applicability, subsequent investigations should integrate clinical data from AD patients, evaluating the diagnostic and molecular stratification tools developed here within large-scale, multicenter trials. Besides, single-cell profiling, although providing detailed resolution, was restricted to a cohort of 9 AD individuals, which may not fully encompass the diverse astrocytic states present across the disease spectrum. Moving forward, the incorporation of advanced AI methods into higher-resolution single-cell and spatial transcriptomic datasets from AD patients, alongside preclinical studies, should enable dynamic monitoring of mitophagy activity in astrocytes during AD progression. In addition, the precise downstream signaling pathways through which ISTN1 in astrocytes influences neuronal integrity via mitophagy remain incompletely defined. Future work should emphasize *in vivo* experimentation employing conditional ITNSN1 knockout or astrocyte-specific overexpression in AD mouse models to establish causality and validate the *in silico* knockout findings. Concurrently, high-throughput screens targeting astrocyte-selective ITSN1 modulators may offer more specific therapeutic alternatives compared with broad-spectrum agents such as statins. Similarly, BRD-K10008415,identified as a potential therapeutic through computational approaches, requires rigorous preclinical validation to assess its efficacy, optimal dosage, blood-brain barrier permeability, and capacity to modulate the proposed MA-associated axis and ITSN1 in living systems.

## Data Availability

The datasets presented in this study can be found in online repositories. The names of the repository/repositories and accession number(s) can be found in the article/Supplementary material.
